# Repurposed Transcriptomic Data Reveal Small Viral RNA Produced by Influenza Virus during Infection in Mice

**DOI:** 10.1371/journal.pone.0165729

**Published:** 2016-10-27

**Authors:** Amanda Koire, Brian E. Gilbert, Richard Sucgang, Lynn Zechiedrich

**Affiliations:** 1 Program in Structural and Computational Biology and Molecular Biophysics, Baylor College of Medicine, Houston, Texas, United States of America; 2 Department of Molecular Virology and Microbiology, Baylor College of Medicine, Houston, Texas, United States of America; 3 Verna and Marrs McLean Department of Biochemistry and Molecular Biology, Baylor College of Medicine, Houston, Texas, United States of America; 4 Department of Pharmacology, Baylor College of Medicine, Houston, Texas, United States of America; University of Georgia, UNITED STATES

## Abstract

Influenza virus, a highly infectious ssRNA virus, replicates in the nucleus of host cells. This unusual feature brings the possibility that the virus may hijack host small noncoding RNA metabolism. Influenza small viral RNA production has been examined *in vitro* but has not yet been studied in an *in vivo* setting. We assessed small RNA species from influenza virus during mouse infection by mining publicly available mouse small RNA transcriptome data. We uncovered 26 nt reads corresponding to svRNA, a small viral RNA previously detected *in vitro* that regulates the transition from transcription to replication during infection, and found a strong positive correlation between svRNA production and host susceptibility to influenza virus infection. We also detected significant overrepresentation of a non-coding 23 nt sequence that we speculate may behave like a miRNA and work with influenza protein NS1 to prevent the transcription and maturation of interferon-stimulated mRNAs.

## Introduction

Influenza A virus is a highly infectious seasonal pathogen responsible for substantial global morbidity and mortality. Annual epidemics affect 5–10% of adults and 20–30% of children, and cause approximately half a million deaths worldwide [1]. The economic impact of this infectious disease is similarly staggering; the total annual economic burden of influenza epidemics in the United States alone is estimated to be over $87 billion considering both direct medical costs and lost earnings [2].

The influenza virus genome is organized into eight single-stranded RNA (ssRNA) segments of negative polarity that code for 11 genes [3–4] (Table 1). After adsorption and entry into the nucleus of the host cell, the genomic viral ssRNA (vRNA) is used as a template to synthesize ssRNA of positive polarity: either messenger RNAs (mRNA) for translation, or full length complementary copies (cRNA) used as intermediates to produce more vRNA (3). The virus alternates between making mRNA and cRNA, ultimately generating either viral proteins or additional progeny virions.

**Table 1:** The eight viral segments of the influenza A genome.

Segment number	Segment length (nt)	Segment Protein(s)	Segment Protein Abbr.	Segment Protein Function(s)	NCBI Reference Sequence Identifier
**1**	2,341	Polymerase basic protein 2	PB2	Initiates RNA synthesis as part of the viral RNA dependent RNA polymerase and performs host mRNA ‘cap-snatching’	NC_002023.1
**2**	2,341	Polymerase basic protein 1	PB1	Initiates RNA synthesis as part of the viral RNA dependent RNA polymerase and performs RNA chain elongation	NC_002021.1
**3**	2,233	Polymerase acidic protein	PA	Initiates RNA synthesis as part of the viral RNA dependent RNA polymerase and is implicated in promoter binding, ‘cap-snatching,’ virus assembly, and proteolysis	NC_002022.1
**4**	1,778	Hemagglutinin	HA	Binds to host cell receptors, facilitates entry of viral genome into host cell	NC_002017.1
**5**	1,565	Nucleoprotein	NP	Encapsidates the viral genome	NC_002019.1
**6**	1,413	Neuraminidase	NA	Destroys sialic-acid containing receptors of host and viral membrane to allow for budding and release of progeny virions from the host cell surface	NC_002018.1
**7**	1,027	Matrix proteins 1 and 2	M1/M2	Matrix protein/ Ion channel	NC_002016.1
**8**	890	Non-structural proteins 1 and 2	NS1/NS2	Host antiviral response inhibitor/ Nuclear export protein	NC_002020.1

The switch from translation to replication during infection is in part regulated by small viral RNAs (svRNAs) 22–27 nucleotides in length [5]. svRNAs are derived from the 5’ untranslated regions (UTRs) of each influenza virus vRNA segment [5], highly conserved regions that also function in genome packaging [6] and polyadenylation [7]. The influenza virus RNA-dependent RNA polymerase (RdRp) is composed of three subunits: the basic proteins PB1, involved in elongation, PB2, involved in cap binding, and the acidic protein, PA. svRNA biases the RdRp of the virus toward replication in a segment-specific manner by associating with the RNA-binding cleft of the polymerase acidic protein (PA) in the polymerase complex [8]. Once in the cleft, the svRNA interacts with the cRNA that matches its segment of origin, and this interaction allows the RdRp to synthesize complete vRNA copies of that segment [8].

Although the presence and function of svRNAs have been studied using multiple different influenza virus strains in cell lines [5,8,9], their generation has not yet been reported in an *in vivo* model. Additionally, unlike other non-retroviral ssRNA viruses, the influenza virus replicates in the host nucleus and is exposed to host nuclear mRNA processing factors, potentially producing other small RNA species during the course of infection. Although the data necessary to explore the question of *viral* miRNA production *in vivo* have existed for years, transcriptome sequencing focused on *host* miRNA responses to influenza infection. Here, we repurposed publically accessible small RNA transcriptome data from influenza A/PR/8/34 (PR8)-infected mice [10] to assess small RNA and svRNA produced by the influenza virus during infection. In addition to establishing that influenza-specific small RNA and *in vivo* production of svRNA could be detected amongst the mouse transcriptome reads, we also found strong support for the svRNA findings produced by cell culture models, as well as additional results apparently unique to the *in vivo* environment.

## Results

### Detection of influenza virus small RNA

To search for potential small RNAs produced by PR8 during infection, we obtained the small RNA transcriptome data produced by Peng *et al*. (2011)[10]. In this work, the investigators infected four different founder strains of mice from the “Collaborative Cross,” a recombinant inbred mouse resource for mapping complex traits [11], with influenza virus strain PR8. Lungs from twelve mice in total were collected for small RNA transcriptome sequencing at two days post infection, producing data from two infected mice and one mock-infected mouse available per strain.

**Fig 1:**
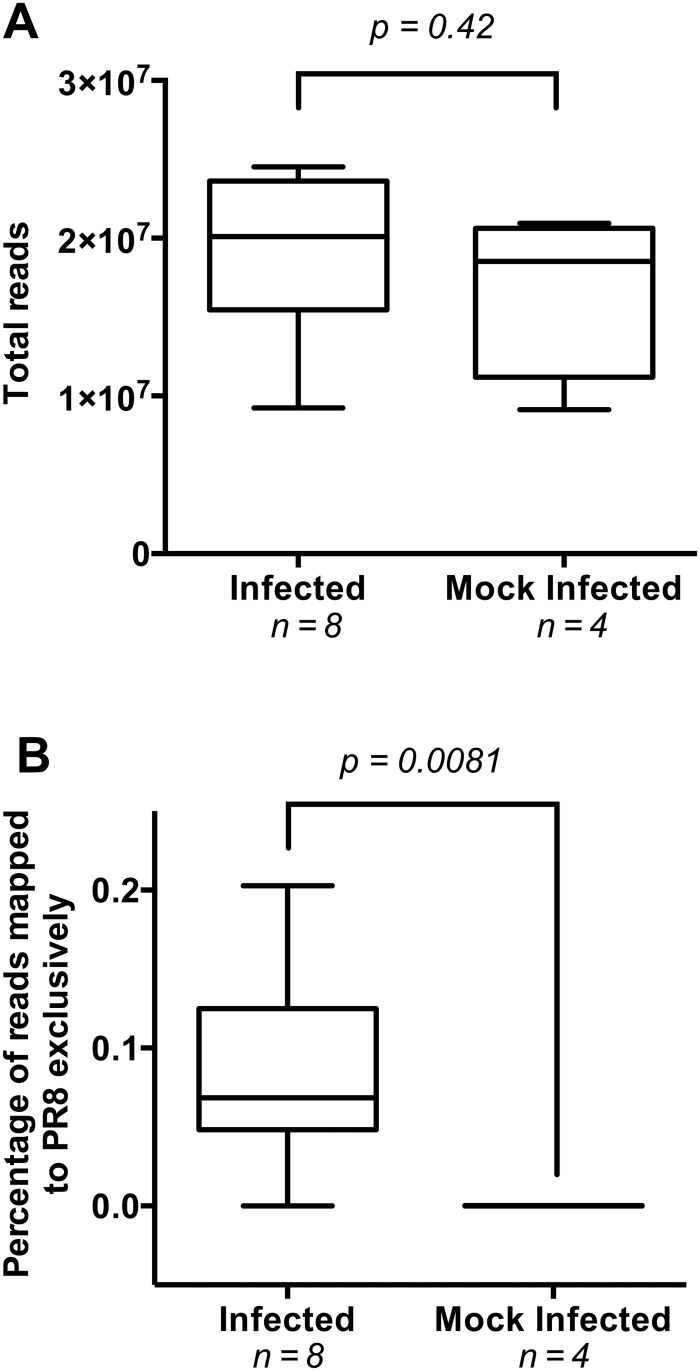
Evaluation of total and PR8-specific reads in infected and mock-infected mice. (A) Total small RNA reads detected in mock-infected and infected mouse lungs. Lung samples were taken at two days post infection with influenza A virus PR8. Small RNAs were purified and sequenced on an Illumina Genome Analyzer IIx system. The infected mouse group contained two replicates from each mouse strain (129, CAST, PWK, WSB) and the mock-infected group contained a single sample from each mouse strain. Groups were compared with a Mann-Whitney test. (B) Percentage of influenza virus-specific reads in each mouse group. Adaptor-trimmed reads mapping uniquely to the PR8 genome are shown as a percentage of total RNA reads. Mock-infected and infected groups were compared with a Mann-Whitney test. The data are presented in a box-and-whiskers plot that uses the centerline to indicate median, the box to indicate quartiles, and the whiskers to indicate range.

The dataset consisted of approximately 219 million adaptor-trimmed reads sized 15–53 nucleotides (nt), corresponding to an average of 18 million reads per sample. Strand information of the reads was preserved. The total number of small RNA reads did not differ significantly between the infected and mock-infected mice (Mann-Whitney test; *p* = 0.42, Fig 1A), indicating that preparation and isolation of small RNA was similarly efficient in all samples. We mapped the RNA reads for each sample to the mouse reference genome C57BL/6J and to the PR8 influenza virus genome. Reads mapping exclusively to the PR8 genome were considered high-confidence influenza virus reads and all subsequent analyses are based on these reads.

The percentage of small RNA reads mapping exclusively to the influenza virus genome was significantly higher in the infected group than the mock-infected group (Mann-Whitney test; *p* = 0.0081, Fig 1B). Of the eight virus-exposed mice, one of the WSB replicates, however, did not appear to have been successfully infected because very few sequencing reads from that animal mapped to the influenza genome; this sample was excluded from subsequent analysis. In Supplementary Data Table 1 from Peng *et al*. 2011 [10], the same mapping paucity for this mouse was noted, but not discussed. For the seven remaining infected mice, exclusively PR8-mapped reads constituted ~0.1% of the total reads, or ~17,000 reads per sample (S1 Table).

### Characterization of influenza small RNA

In the infected samples, vRNA was present at a significantly (*p* = 0.0007) higher abundance than cRNA (S1 Table). The influenza virus-specific reads were not evenly distributed among the segments, with segments 5, 2, and 6 accounting for an average of ~30%, ~22%, and ~15% of the total reads, respectively (S2 Table). This uneven distribution was not explained by inherent differences in the sizes of the segments, which range from 890 to 2,341 nt in length—the average percentage of total reads attributed to a segment did not correlate significantly to the segment length (*R*^2^ = 0.169, S1 Fig).

**Fig 2:**
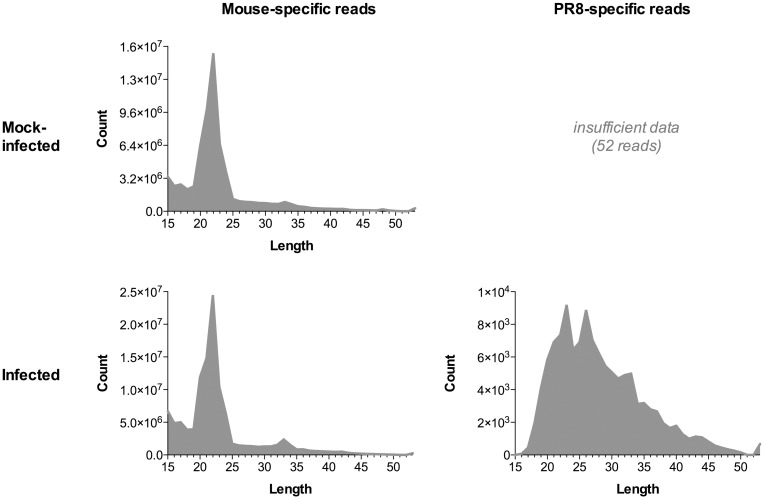
Read length distribution of mouse and influenza virus-specific small RNA reads. Adapter-trimmed small RNA reads from all samples were separated based on whether they originated from a mock-infected or infected mouse, and whether they mapped uniquely to the mouse genome or to the PR8 genome. For each of these categories, small RNA reads were combined and assessed for the distribution of their read lengths.

The read length distribution of the virus-specific small RNA reads (Fig 2A) revealed two peaks at 23 and 26 nt, both of which fall within the 22–27 nt range for svRNA described in Perez *et al*. 2010 [5]. In contrast to the influenza-derived small RNA reads, the length distribution of mouse-derived small RNA reads from mock-infected mice revealed only a single peak at 22 nt (Fig 2B), indicating that the overall profiles of influenza and mouse small RNA species are distinct. The mouse-derived small RNA peak that we identified at 22 nt is consistent with the original analysis performed on these hosts (see S1 Fig of Peng *et al*. 2011) [10].

### Characterization of influenza svRNA

**Fig 3:**
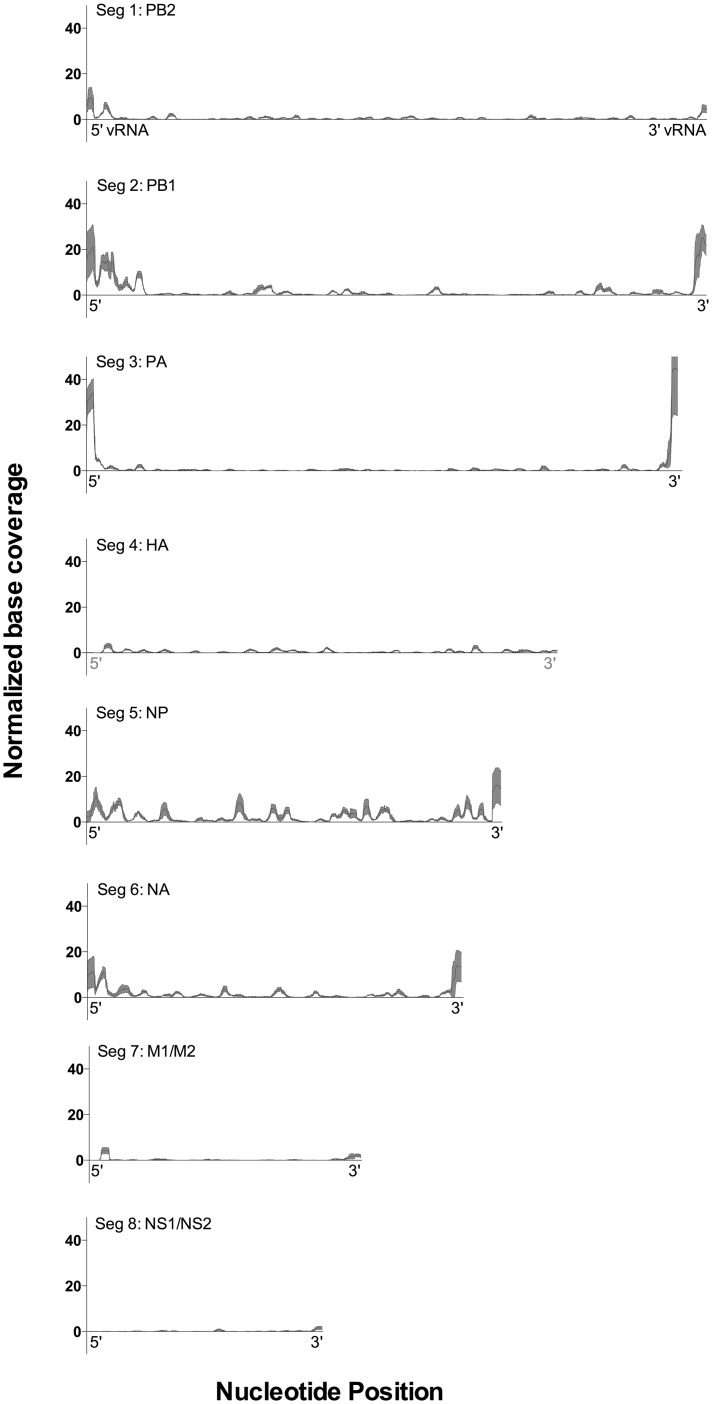
Segment-specific genome coverage of reads mapped to PR8. Influenza virus-specific reads were sorted by genomic coordinate. Strand-specific coverage of each base in the reference sequence was quantified and base coverage values for the seven infected mouse samples were normalized for the total number of PR8-specific reads in the respective sample and displayed as base coverage per thousand reads. For each position in the PR8 genome, the mean and 95% CI of the normalized coverage of the vRNA were calculated and are displayed as black and red, respectively. Nucleotide position is counted from the 5’ end of the vRNA.

**Fig 4:**
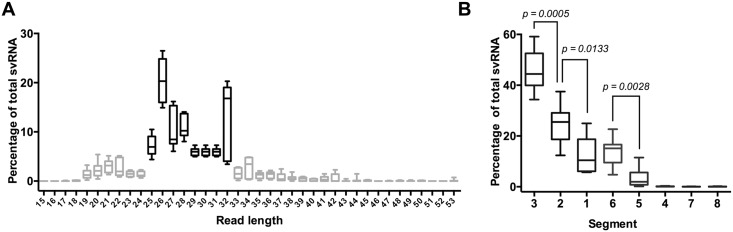
Size and segment distributions of svRNA reads. (A) Percent of total svRNA attributed to each read length. (B) Percent of total svRNA attributed to each segment. Segments were sorted from most represented to least represented in the svRNA and segment percentages were compared with unpaired t-tests.

Assessing strand-specific coverage of the genome, we detected high levels of coverage for the 5’ end of the vRNA strand for several segments, indicating the presence of svRNA *in vivo* (Fig 3). Across the seven infected mice, an average of 7.3% of all influenza virus-specific reads could be attributed to svRNA. Across all experiments, 78% of the svRNA reads were 25–32 nt long (Fig 4A); reads lengths of 15–24 nt and 33–55 nt comprised the remainder of the svRNA. The svRNA reads were significantly enriched for 26 nt reads in particular (chi square test; *p* < 0.0001). svRNA contributes 21% of all PR8-specific reads 26 nt in length, suggesting that the peak at 26 nt in Fig 2 can be attributed to the presence of influenza virus svRNA.

**Fig 5:**
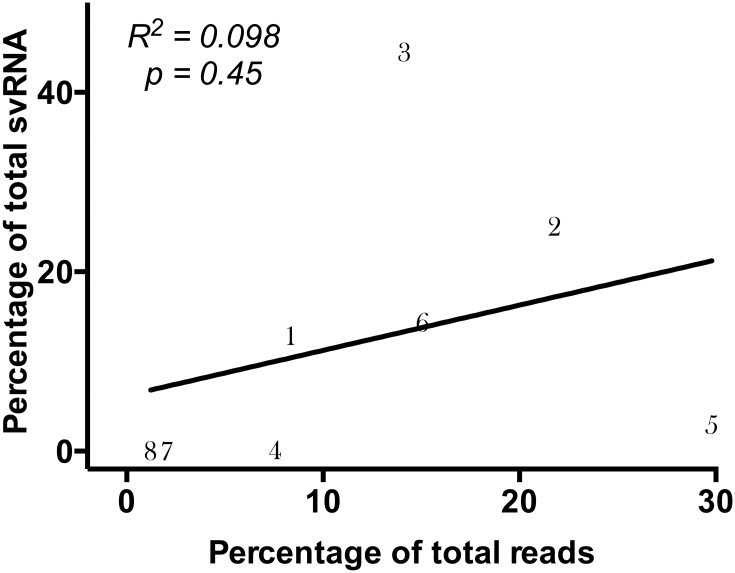
Correlation between segment read abundance and segment svRNA abundance. The percentage of total influenza-specific reads attributed to each segment, as well as the percentage of total svRNA reads attributed to the segment, was calculated across all infected mouse reads. Each segment is represented numerically on the graph, with the line representing the linear regression of the data.

The vast majority of svRNA (96.7%) mapped to segments 1, 2, 3, and 6 (S3 Table). In any given sample, approximately half the svRNA reads were derived from segment 3 (PA), which was significantly more abundant than the next most common source for svRNA, segment 2 (*p* = 0.0005; Fig 4B). The fraction of total reads attributed to each segment did not correlate with the percentage of total svRNA attributed to the segment (*R*^2^ = 0.098; Fig 5). This result indicates that these reads are unlikely to be random breakdown fragments of longer RNAs from the same segment and that the overrepresentation of svRNA from certain segments is unlikely to be from the virion inocula.

### Correlation between host susceptibility to influenza and svRNA production

**Fig 6:**
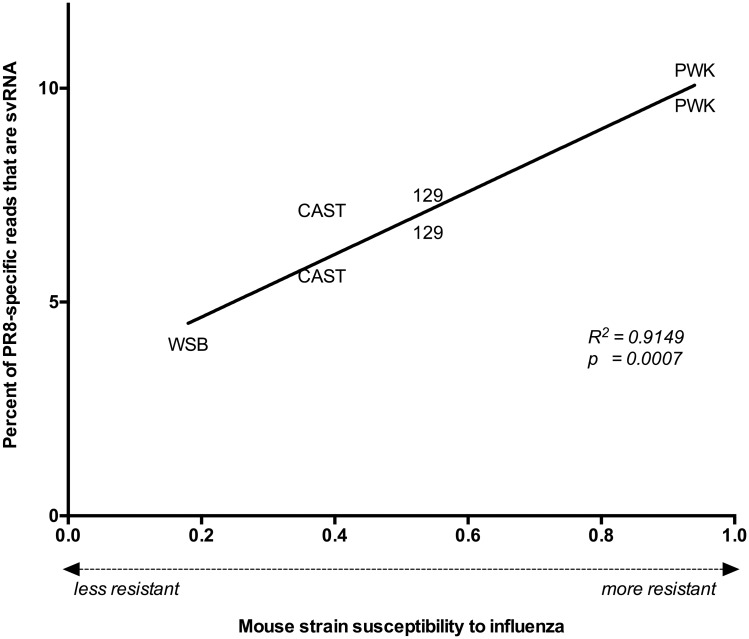
Relationship between host immunity and svRNA abundance. For each infected mouse, the susceptibility of the mouse strain to influenza virus was plotted against the abundance of svRNA in the PR8 reads for that sample. Lower values indicate that the mouse strain is more susceptible to PR8 (high viral titer, inflammation and weight loss); higher values indicate that the mouse strain is more resistant to PR8 (low viral titer, little inflammation, and weight loss). Pearson’s linear correlation was applied to the dataset to quantify the relationship between the variables, and the line connecting the data points represents the linear regression of the data.

The four mouse strains used in the experiments of Peng *et al*. (129, CAST, PWK, WSB) were chosen because they represent a wide range of susceptibility to infection with PR8 [12–14]. We found that the percentage of svRNA in the PR8-specific reads correlated significantly to the Mouse Phenome Database "susceptibility" phenotype of the mouse strains to influenza virus infection (*p* = 0.0007; Fig 6) [12–13], with more resistant mouse strains having a higher svRNA fraction in their PR8-specific reads. The Pearson’s linear correlation of this relationship was 0.9565 [0.7273 to 0.9938; 95% confidence interval (CI)], with an associated coefficient of determination indicating that >91% of the differences in svRNA burden amongst different mice can be linked to the susceptibility of the mouse strain to influenza virus infection. svRNA that is specifically 26 nt in length, and the main component of the influenza-derived read peak seen in Fig 2, is also significantly correlated to susceptibility (*R*^2^ = 0.70; *p* = 0.02). In contrast, total influenza-derived small RNA burden, represented by the percentage of PR8-specific small RNA reads in each sample, does not correlate with susceptibility (*R*^2^ = 0.12; *p* = 0.45). These data demonstrate that svRNA in particular, rather than influenza small RNA in general, exhibits a strong relationship with the influenza susceptibility phenotype.

### Characterization of the 23 nt peak

Although svRNA enrichment explained the 26 nt peak in the influenza small RNA profile, svRNA reads composed only 1.4% of the 23 nt reads, and therefore does not account for the second peak observed at 23 nt (Fig 2). Across all samples, 24.5% of the 23 nt reads mapped to a region corresponding to the terminal nucleotides in the 3’ UTR of the segment 3 (PA) vRNA strand; this particular sequence is dramatically overrepresented amongst 23 nt reads (*p* < 0.001; chi square test). Its abundance is 33-fold higher than the next most represented sequence, and 42-fold higher than the most abundant svRNA sequence in the peak. Though sometimes mapped as a part of longer reads derived from the same region, the terminal 3’ end of the segment 3 vRNA strand was identified specifically as a 23 nt sequencing read approximately 40% of the time; a highly nonrandom occurrence (chi square test; *p* < 0.001) that indicates a preference for cleavage to this particular length and region. Approximately 5% of the reads in the 23 nt peak are derived from the vRNA 3’ UTR from segments 2 and 6; these sequences are also overrepresented amongst 23 nt reads (*p* < 0.001). Whereas the 26 nt peak can be attributed to an overrepresentation of reads from the 5’ UTR of the vRNA, the 23 nt peak is derived from an abundance of reads from the 3’ UTR of the vRNA.

Although levels of 3’UTR vRNA from segment 3 are high, abundance of the specifically derived 23 nt species did not correlate with mouse infection susceptibility (p = 0.83). We hypothesized that this species could be a previously undetected short RNA that serves a function during influenza infection. Given that 23 nt is close to the ~22 nt size described for miRNA [15], we speculate that this small RNA may modulate translational repression of specific and relevant host proteins. In order to understand what processes this small RNA might affect if it does in fact behave as a miRNA, we predicted miRNA targets for the 23 nt sequence using three methods: miRDB [16], TargetScan 5 [17], and DIANA MR-microT [18]. Thirty-three genes were agreed upon as targets by at least two of the three methods (Table 2, S4 Table), and the list of targets was enriched for genes annotated to be involved in the ‘Formation and Maturation of mRNA Transcript’ according to Pathway Commons (*q* = 0.0008) [19]. Considering that the part of the putative miRNA essential for binding to the target mRNA (the seed region) is highly dissimilar to the seed regions from equivalent 3’ UTR 23 nt sequences on the other vRNA segments (S2 Fig), the overrepresentation of segment 3 in particular indicates that specific downregulation of these genes may be beneficial to viral activity.

**Table 2:** Target predictions for overrepresented segment 3 vRNA sequence.

	miRNA target prediction method		
	mirDB	DIANA MR-microT	TargetScan 5
USP3	***√***	***√***	***√***
ADAMTS6	***√***	***√***	***√***
NUP153	***√***	***√***	***√***
B4GALT1	***√***	***√***	***√***
H3F3B	***√***	***√***	***√***
SLITRK5	***√***	***√***	***√***
RPRD1A	***√***	***√***	*x*
UNC79	***√***	***√***	*x*
ERC2	***√***	***√***	*x*
SYT15	***√***	***√***	*x*
ATM	***√***	***√***	*x*
CSN3	***√***	***√***	*x*
EPHA7	***√***	***√***	*x*
HMGXB4	***√***	***√***	*x*
ELAVL4	***√***	***√***	*x*
CTTN	***√***	*x*	***√***
VGLL3	***√***	*x*	***√***
PLEKHA6	***√***	*x*	***√***
TCEA1	***√***	*x*	***√***
CDC40	***√***	*x*	***√***
ARID5B	***√***	*x*	***√***
CPEB2	***√***	*x*	***√***
PCBP1	*x*	***√***	***√***
FOXN3	*x*	***√***	***√***
GPBP1	*x*	***√***	***√***
CDH2	*x*	***√***	***√***
GJC1	*x*	***√***	***√***
SP1	*x*	***√***	***√***
SLC6A14	*x*	***√***	***√***
KLHL14	*x*	***√***	***√***
NEGR1	*x*	***√***	***√***
ANKRD49	*x*	***√***	***√***
EGLN3	*x*	***√***	***√***

## Discussion

*In vitro*, influenza virus svRNA is generated abundantly at the transition from viral transcription to replication [5]. Infection by multiple influenza A virus subtypes in cell lines derived from multiple mammalian species can produce svRNA [5]. Ours is the first report of the production of influenza virus svRNA *in vivo*.

Our findings support a number of hypotheses put forth based on previous *in vitro* work. First, the small RNA transcriptomes of infected *in vivo* lung samples, harvested at a time point twelve hours later than any *in vitro* experiment, confirm that svRNA production continues late into the infection process. Curiously, the predominant length of svRNA detected *in vivo*, 26 nt, is different from the predominant lengths reported *in vitro* by Perez *et al*. (25 nt and 27 nt) [5] and Umbach *et al*. (19, 21, and 22 nt) [9]. In both these earlier reports, as well as ours, substantial variation in svRNA length within datasets is interpreted to indicate that svRNA production is a somewhat stochastic process. Stochasticity may also contribute to heterogeneity across datasets, although factors such as influenza virus strain [9] and method used to determine svRNA length [5] seem to affect the reported length as well. In this particular case, the later time point in the infection process and the *in vivo* environment are also plausible explanations for the length differences.

Second, although the precise sizes of the svRNA differ, the *in vivo* findings replicated the observation that the predominant svRNA length is segment-dependent (S3 Table) [5, 9], and that segments 4, 7, and 8 are relatively minor sources of svRNA [5, 9]. The variation among segments in the amount of svRNA produced *in vivo* also supports the understanding that each segment produces svRNA independently, possibly to maintain the stoichiometric balance of segments during the creation of new virions.

Finally, the absence of a significant correlation between the fraction of reads and the fraction of svRNA attributed to a given segment supports that svRNA is more likely to be synthesized from a cRNA template than produced by targeted cleavage of full-length vRNA transcripts.

In addition to providing an *in vivo* comparison for previous *in vitro* findings, we also uncovered results unique to the *in vivo* environment. The *in vivo* data linked svRNA burden and host susceptibility, a relationship that could not be detected in *in vitro* experiments. This strong linear relationship may relate the timing and frequency of transcription to replication switching to the pressures exerted by the host immune system. Whether the host or the influenza virus is directly responsible for the mechanism producing the elevated svRNA levels is not known. Perhaps resistance of the specific mouse strain to viral infection drives extended viral replication to maintain its presence in the host leading to elevated svRNA levels. Though beyond the scope of the repurposed data, this possibility warrants further exploration. Furthermore, whereas Umbach *et al*. noted a modest enrichment for reads originating from the 3’ end of the vRNA in the PR8 infection of cell culture [9], the abundance of 23 nt reads derived from the 3’ UTR vRNA we found is apparently specific to the mouse infection environment.

Further study is necessary to determine whether this small 23 nt RNA behaves in a miRNA-like fashion, but computationally it is predicted to influence the host immune response by mimicking some of the actions of the viral NS1 protein. Amongst its diverse strategies for antagonizing host immunity, the NS1 protein inhibits transcriptional elongation by binding to PAF1, causing a PAF1 deficiency at transcriptional end sites and resulting in selective inhibition of antiviral gene expression [20]. Inhibition of pre-mRNA splicing is also accomplished by NS1 associating with the spliceosomes and blocking subsequent catalytic steps of splicing; cellular pre-mRNAs can then be accessed by the viral cap-dependent endonuclease to produce the capped primers required for viral mRNA synthesis [21]. Three of the 33 genes predicted to be targets of the putative viral miRNA participate in these same processes.

RPRD1A, the second highest ranked target predicted by miRDB, binds to RNA polymerase II and participates in the dephosphorylation of key residues in its CTD region, thus allowing for the transition from transcription initiation to elongation [22]. TCEA1, also known as TFIIS, not only independently facilitates transcription elongation but also directly binds and demonstrates synergy with PAF1, a known target of the influenza virus NS1 protein [23]. CDC40 is responsible for the second step of pre-mRNA splicing, in which the 3’ splice site is cleaved and the exons are joined to create a mature mRNA [24]. Repression of this gene could mimic the effect of NS1 by targeting the catalytic step of pre-mRNA processing directly and causing the retention of cellular pre-mRNAs in the host cell nucleus.

Although the functions of this small RNA must remain speculative, a virally derived miRNA could affect multiple aspects of infection physiology. The sequencing protocol used to produce the data for this study was limited to RNAs < 54 nt in length and with 3’OH and 5’ modifications such as those processed by Dicer and Drosha; we are thus unable to assess the relationship between the levels of the small 23 nt RNA and the expression of its putative targets. Pairing small RNA sequencing with an approach to capture host gene expression changes would be an important next step.

In conclusion, repurposing mouse lung transcriptomic data in the setting of influenza infection was a successful strategy for identifying influenza-specific small RNA and *in vivo* production of svRNA. We found strong support for results performed in cell culture, and discovered a small RNA meriting further investigation.

## Materials and Methods

### Retrieval of source data

Source data regarding small RNA transcriptomes were derived from Peng *et al*. 2011 [10]. In summary, ten-week-old mice of four different strains (129, WSB, PWK, and CAST) were infected intranasally with either phosphate-buffered saline or with 500 PFU of influenza A virus strain A/PR/8/34 (H1N1; PR8). The small RNA transcriptome was sequenced two days post-infection for twelve mice total; two PR8-infected lung samples from each mouse strain as well as one control mouse lung sample from each strain mock-infected with saline were processed. This sequencing process preserved the strand information of the resulting reads. Raw sra files of the small RNA transcriptomes obtained by Peng *et al*. 2011 [10] were downloaded from the NCBI Gene Expression Omnibus (GEO), accession identifier GSE36971. Source data regarding influenza virus (H1N1; PR8) susceptibility values (based on multiple experimental findings) for the mouse strains used in the experiments were obtained though the Mouse Phenome Database [12–13].

### Adapter trimming

Sra files were converted to fastq format with sratoolkit v2.3.2–5. The 3′ adapter (5′ TGGAATTCTCGGGTGCCAAGG) was removed with Cutadapt v1.2.1 [25]. Reads < 15 nt in length after adapter trimming were discarded, as they map nonspecifically to the mouse genome.

### Read mapping

The remaining short RNA reads were aligned to the mouse reference genome (mm10, Dec 2011, GRCm38, reference strain C57BL/6J) with Bowtie2 v2.1.0 [26] using the ‘—very-sensitive’ setting. Reads that did not align to the mouse reference genome were subsequently aligned to the influenza virus PR8 reference genome (GenBank accession no. AF389115 to AF389115), again using the ‘—very-sensitive’ setting to identify influenza virus-specific reads.

### svRNA isolation

svRNA was defined as reads with at least 15 bp overlapping with the svRNA sequences put forth by Perez *et al*. 2010 [5]. Reads meeting these specifications were removed by Cutadapt and isolated for additional analysis.

### Genome coverage mapping

Sam files of influenza virus-specific reads were converted to bam format and sorted by coordinate using the Picard tools package (http://picard.sourceforge.net). Strand-specific coverage of each base in the reference sequence was quantified using the BEDTools suite [27]. Base coverage values for the infected mouse samples were normalized for the amount of total coverage in the respective sample. For each position in the PR8 genome, the mean and 95% CI of the normalized coverage were calculated using Prism 6 software.

### miRNA target prediction and analysis

miRNA target prediction was performed by miRDB [16], TargetScan 5 Custom [17], and DIANA MR-microT [18]. Targets that were detected by at least two of the three prediction methods were considered to be of interest. For miRDB and DIANA MR-microT, which report reliability scores for the targets on a continuous scale, thresholds for detection were set as score > 80 for miRDB (as recommended by the miRDB server), and as positive predictive value > 0.8 for DIANA MR-microT. Hypergeometric gene set enrichment analysis (GSEA) of the miRNA target predictions against Pathway Commons was performed by WebGestalt [28].

## Supporting Information

S1 Fig Correlation between segment length and read abundance. For each infected mouse, the percentage of total influenza-specific reads attributed to each segment was calculated. The mean and error for each segment is displayed, with the line representing the linear regression of the data.
(TIFF)

S2 Fig Terminal 23 nt sequences of all vRNA segments. The terminal 23 nt of the 3’ UTR are represented for each vRNA segment; the potential miRNA ‘seed regions’ of these sequences are annotated as nt 2–8.
(TIFF)

S1 Table Read mapping of small RNA to PR8 genome. (PDF)

S2 Table Segment distribution of PR8-specific reads. (PDF)

S3 Table Length and segment distribution of influenza virus svRNA sequences. (PDF)

S4 Table miRNA target predictions for 5'-TTGGATCAGTACCTGCTTTCGCT-3'. (PDF)
