# Correction: Repurposed Transcriptomic Data Reveal Small Viral RNA Produced by Influenza Virus during Infection in Mice

**DOI:** 10.1371/journal.pone.0189384

**Published:** 2017-12-05

**Authors:** Amanda Koire, Brian E. Gilbert, Richard Sucgang, Lynn Zechiedrich

The following information is missing from the Funding section: This work was supported by RP160283—Baylor College of Medicine Comprehensive Cancer Training Program.
