# Supplementary figures and images for: Repurposed Transcriptomic Data Reveal Small Viral RNA Produced by Influenza Virus during Infection in Mice
Source: PLoS One. 2016 Oct 27;11(10):e0165729. doi: 10.1371/journal.pone.0165729 (PMC5082947; doi:10.1371/journal.pone.0165729)

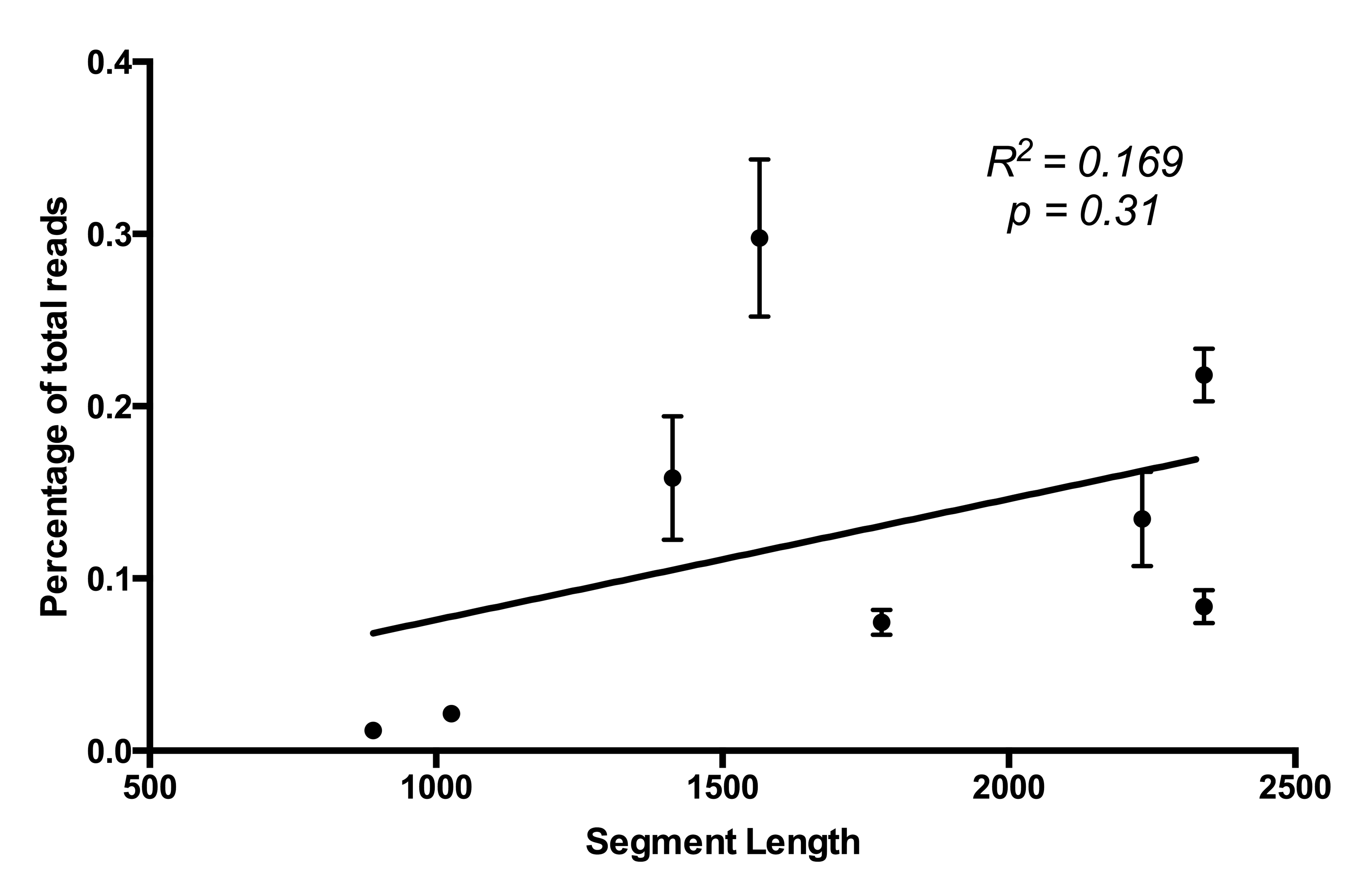

Supplement: S1 Fig — For each infected mouse, the percentage of total influenza-specific reads attributed to each segment was calculated. The mean and error for each segment is displayed, with the line representing the linear regression of the data. (TIFF) [file pone.0165729.s001.tiff]
