# Supplementary material for: Repurposed Transcriptomic Data Reveal Small Viral RNA Produced by Influenza Virus during Infection in Mice
Source: PLoS One. 2016 Oct 27;11(10):e0165729. doi: 10.1371/journal.pone.0165729 (PMC5082947; doi:10.1371/journal.pone.0165729)
Supplement: S1 Table — (PDF) [file pone.0165729.s003.pdf]

Table S1: Read mapping of small RNA to PR8 genome

Expt (SRA identifier)	Mouse Strain	Strain susceptibility to influenza	Total reads	PR8-specific reads	% PR8-specific reads	cRNA	vRNA	total svRNA	% svRNA of PR8-specific reads	% svRNA of total reads
SRR452392	129	0.54	20005573	40566	0.20	15539	25027	3040	7.49	0.015
SRR452393	129	0.54	16086702	11234	0.07	4635	6599	744	6.62	0.005
SRR452395	CAST	0.38	15252479	19540	0.13	8679	10861	1095	5.60	0.007
SRR452396	CAST	0.38	9247648	10668	0.12	4156	6512	761	7.13	0.008
SRR452398	PWK	0.94	24519470	15047	0.06	6316	8731	1443	9.59	0.006
SRR452399	PWK	0.94	20194273	8889	0.04	3556	5333	926	10.42	0.005
SRR452402	WSB	0.18	22905860	15389	0.07	8519	6870	615	4.00	0.003
SRR452401	WSB*	0.18	23873659	26	1.09E-04	13	13	-	-	-
*average***	-	-	18316001	17333	0.10	7343	9990	1232	7.27	0.007
SRR452394	129 mock-infected	0.54	20939618	44	2.10E-04	22	22	-	-	-
SRR452397	CAST mock-infected	0.38	9135927	5	5.47E-05	3	2	-	-	-
SRR452400	PWK mock-infected	0.94	17364761	1	5.76E-06	0	1	-	-	-
SRR452403	WSB mock-infected	0.18	19712916	2	1.01E-05	0	2	-	-	-

* indicates a replicate injected with PR8 that does not appear to have been successfully infected
**average for infected examples excludes abnormal sample SRR452401
