# Supplementary material for: Repurposed Transcriptomic Data Reveal Small Viral RNA Produced by Influenza Virus during Infection in Mice
Source: PLoS One. 2016 Oct 27;11(10):e0165729. doi: 10.1371/journal.pone.0165729 (PMC5082947; doi:10.1371/journal.pone.0165729)
Supplement: S2 Table — (PDF) [file pone.0165729.s004.pdf]

Table S2: Segment distribution of PR8-specific reads

	SRR452392	SRR452393	SRR452395	SRR452396	SRR452398	SRR452399	SRR452402	**Total**	% of all PR8-specific reads
Segment 1	3468	1023	1515	1055	1143	630	1322	**10156**	8.37
Segment 2	8798	2426	4652	2479	3007	2009	3048	**26419**	21.77
Segment 3	7119	1844	1838	1276	1934	1172	1966	**17149**	14.13
Segment 4	3113	805	1421	675	1212	630	1318	**9174**	7.56
Segment 5	11119	2859	7269	3360	3944	2352	5248	**36151**	29.79
Segment 6	5586	1806	2272	1521	3204	1787	2107	**18283**	15.07
Segment 7	799	288	382	218	381	218	235	**2521**	2.08
Segment 8	564	183	191	84	222	91	145	**1480**	1.22
**Total**	**40566**	**11234**	**19540**	**10668**	**15047**	**8889**	**15389**	**121333**	
