# Supplementary material for: Repurposed Transcriptomic Data Reveal Small Viral RNA Produced by Influenza Virus during Infection in Mice
Source: PLoS One. 2016 Oct 27;11(10):e0165729. doi: 10.1371/journal.pone.0165729 (PMC5082947; doi:10.1371/journal.pone.0165729)
Supplement: S3 Table — (PDF) [file pone.0165729.s005.pdf]

Table S3: Length and segment distribution of influenza virus svRNA sequences

	Seg 1	Seg 2	Seg 3	Seg 4	Seg 5	Seg 6	Seg 7	Seg 8	*Total*	*% all svRNA*
15	0	0	0	0	0	0	0	0	0	0.00
16	0	0	0	0	0	0	0	0	0	0.00
17	1	0	0	0	0	0	0	0	1	0.01
18	3	0	0	0	0	0	0	1	4	0.05
19	100	0	3	0	0	3	0	0	106	1.23
20	100	0	34	0	0	48	0	0	182	2.11
21	186	0	39	3	0	14	0	1	243	2.82
22	191	27	20	1	0	23	2	1	265	3.07
23	54	30	31	0	1	16	0	0	132	1.53
24	24	46	35	0	1	15	0	0	121	1.40
25	93	295	209	4	0	55	0	0	656	7.61
26	257	153	914	2	0	501	0	0	1827	21.19
27	50	161	541	0	2	158	0	0	912	10.58
28	7	104	749	2	2	94	0	0	958	11.11
29	15	142	245	1	1	116	0	0	520	6.03
30	3	124	227	0	4	58	0	0	416	4.82
31	2	212	51	0	2	113	0	0	380	4.41
32	2	526	527	0	4	5	0	0	1064	12.34
33	7	54	64	0	1	5	0	0	131	1.52
34	0	173	42	0	15	6	0	0	236	2.74
35	1	59	28	0	2	1	0	0	91	1.06
36	1	29	44	0	26	0	0	0	100	1.16
37	0	8	15	0	45	0	1	0	69	0.80
38	1	4	9	0	38	0	0	0	52	0.60
39	3	8	1	0	16	0	0	1	29	0.34
40	1	6	0	0	3	0	0	0	10	0.12
41	0	0	1	0	29	1	0	0	31	0.36
42	1	0	1	0	38	0	0	0	40	0.46
43	0	0	2	0	3	0	0	0	5	0.06
44	0	0	1	0	16	0	0	0	17	0.20
45	4	0	0	0	1	3	0	0	8	0.09
46	0	0	0	0	0	0	0	0	0	0.00
47	0	0	0	0	1	0	0	0	1	0.01
48	2	0	0	0	0	1	0	0	3	0.03
49	0	0	0	0	0	2	0	0	2	0.02
50	0	0	2	0	1	0	0	0	3	0.03
51	0	0	0	0	0	0	0	0	0	0.00
52	0	0	0	0	0	0	0	0	0	0.00
53	2	0	2	0	4	1	0	0	9	0.10
Total	1111	2161	3837	13	256	1239	3	4	**8624**	
% all svRNA	12.88	25.06	44.49	0.15	2.97	14.37	0.03	0.05		
