# Supplementary material for: Repurposed Transcriptomic Data Reveal Small Viral RNA Produced by Influenza Virus during Infection in Mice
Source: PLoS One. 2016 Oct 27;11(10):e0165729. doi: 10.1371/journal.pone.0165729 (PMC5082947; doi:10.1371/journal.pone.0165729)
Supplement: S4 Table — (PDF) [file pone.0165729.s006.pdf]

Table S4: miRNA target predictions for 5'-TTGGATCAGTACCTGCTTTCGCT-3'

Gene	Protein description	mirDB*	DIANA MR-microT**	TargetScan 5
USP3	Ubiquitin carboxyl-terminal hydrolase 3	99	0.96	y
ADAMTS6	a disintegrin-like and metalloprotease (reprolysin type)	93	0.99	y
NUP153	nucleoporin 153	92	0.95	y
B4GALT1	Beta-1,4-galactosyltransferase 1	90	0.94	y
H3F3B	Histone H3.3	86	0.95	y
SLITRK5	SLIT and NTRK-like protein 5 Precursor	91	0.8	y
RPRD1A	Regulation of nuclear pre-mRNA domain-containing protein 1A	98	0.87	nd
UNC79	Component of the NALCN sodium channel complex	97	0.82	nd
ERC2	ERC protein 2	95	0.86	nd
SYT15	Synaptotagmin-15	95	0.9	nd
ATM	Serine-protein kinase ATM	95	0.97	nd
CSN3	Kappa-casein Precursor	92	0.96	nd
EPHA7	Ephrin type-A receptor 7 Precursor	88	0.82	nd
HMGXB4	HMG box domain containing 4	88	0.97	nd
ELAVL4	ELAV-like protein 4	84	0.89	nd
CTTN	Src substrate cortactin	97	*0.74*	y
VGLL3	Transcription cofactor vestigial-like protein 3	93	nd	y
PLEKHA6	Pleckstrin homology domain-containing family A member 6	87	nd	y
TCEA1	Transcription elongation factor A protein	83	*0.61*	y
CDC40	Pre-mRNA-processing factor 17	83	*0.7*	y
ARID5B	AT-rich interactive domain-containing protein 5B	82	nd	y
CPEB2	Cytoplasmic polyadenylation element-binding protein 2	82	*0.51*	y
PCBP1	Poly(rC)-binding protein 1	*67*	0.98	y
FOXN3	Forkhead box protein N3	nd	0.95	y
GPBP1	Vasculin (GC-rich promoter-binding protein 1)	*65*	0.94	y
CDH2	Cadherin-2 Precursor	*72*	0.93	y
GJC1	Gap junction gamma-1 protein	*50*	0.89	y
SP1	Transcription factor Sp1	nd	0.87	y
SLC6A14	Sodium- and chloride-dependent neutral and basic amino acid transporter	nd	0.84	y
KLHL14	kelch-like 14	nd	0.83	y
NEGR1	Neuronal growth regulator 1 Precursor	nd	0.82	y
EGLN3	Hypoxia-inducible factor prolyl hydroxylase 3	nd	0.82	y
ANKRD49	Ankyrin repeat domain-containing protein 49	nd	0.81	y

* mirDB target score
** positive predictive value
